# This is pain

**DOI:** 10.4103/0019-5545.44758

**Published:** 2008

**Authors:** Sanju George

**Affiliations:** Consultant and Senior Research Fellow in addiction psychiatry, Birmingham and Solihull Mental Health NHS Trust, Birmingham, B37 7UR, England. E-mail: sanju.george@bsmht.nhs.uk

Have I told you how much it hurts?

I can't cough but I have to

I can't breathe but I want to

I can't move, it makes me sad

People's pity I hate, your kind words I dislike

This is pain.

Have I told you how much it hurts?

Hoped pills would help but didn't

Wished sleep would but couldn't

Beyond hope, beyond reason

Alive but dead, dead but not gone

In anger I ask, why me?

This is pain.

Have I told you how much it hurts?

In pain I cry

In pain I'm all alone

Clouds of misery hover above me

Angels in white smile at me

Into a distant world I fly

I long to leave

Away from the pain forever.

Dr. Sanju George, MRCPsych, a published poet, educator, and academic, is Consultant and Senior Research Fellow in addiction psychiatry.

[ED.: * An account of the physical, affective, and cognitive aspects of chronic pain, pertains to a patient with terminal lung cancer describing his angst to the psychiatrist.*]

